# Adverse Childhood Experiences and Health Outcomes Among Transition-Age Autistic Youth

**DOI:** 10.1007/s10803-024-06401-7

**Published:** 2024-05-21

**Authors:** Wei Song, Kiley J. McLean, Jordan Gifford, Hailey Kissner, Rosalind Sipe

**Affiliations:** https://ror.org/04bdffz58 grid.166341.7 0000 0001 2181 3113 A.J. Drexel Autism Institute, Drexel University, 3020 Market Street, Suite 560, Philadelphia, PA 19104 USA

**Keywords:** Transition-age autistic youth, Adverse childhood experiences, Mental health, Health outcomes

## Abstract

**Background:**

Adverse childhood experiences (ACEs) have been associated with poor health outcomes in the general population. However, their impact on autistic youth remains unclear.

**Objective:**

The primary objective was to understand how childhood adversity is related to the general health, mental health, and physical health of transition-age autistic youth.

**Participants and Setting:**

Using data from the 2018–2021 National Survey of Children’s Health, this cross-sectional study involved 2056 autistic youth aged 12–17.

**Methods:**

Logistic regression was employed to test the association between three measures of ACEs - individual ACEs, cumulative ACEs, and grouped ACEs based on contexts, and health outcomes of autistic youth.

**Results:**

Our study observed a high prevalence of ACEs among autistic youth, with a substantially higher proportion experiencing multiple ACEs than their neurotypical peers. Individual ACEs were significantly associated with specific health issues. Cumulative ACEs demonstrated a clear dose-response relationship with health outcomes, with higher ACE counts increasing the likelihood of experiencing poor general health, mental health conditions, and physical health issues. Moreover, grouped ACEs associated with health differently, with community-based ACEs being particularly linked to general health status, mental health conditions, and physical health conditions, while family-based ACEs correlated more with more severe mental health conditions and being overweight.

**Conclusion:**

These findings collectively emphasize the importance of addressing ACEs as a public health concern among transition-age autistic youth, highlighting the need for targeted interventions, prevention strategies, and support services to mitigate the negative impact of ACEs on the overall well-being of this growing community.

## Introduction

Adverse childhood experiences (ACEs) refer to some of the most intense stress sources that children can be exposed to, including child maltreatment (e.g., physical, sexual, and emotional abuse) and household dysfunction (e.g., domestic violence, parental separation, caregiver incarceration) (CDC, 2023; Felitti et al., 1998). ACEs are relatively common, with prevalence estimates of the United States (US) population under age 18 having at least one ACE ranging from 48 to 61% (Merrick et al., 2019). Youth with a diagnosis of autism spectrum disorder (ASD) (hereafter referred to as autistic youth) are at greater risk for ACEs compared to their non-autistic peers (Berg et al., 2016; Hoover & Kaufman, 2018; Kerns et al., 2017; Rigles, 2017). This propels research to understand how these adverse events impact health and well-being in the autistic population.

Although the impact of ACEs on autistic children is unclear, we know from studies of children in the general population that cumulative childhood adversity is associated with an increased risk of physical health, mental health, and developmental conditions (Hughes et al., 2017; Bellis et al., 2019; Petruccelli et al., 2019). For example, a systematic review of 37 studies reported that having multiple ACEs is a major risk factor for depression, anxiety, obesity, heart disease, overall poor self-rated health, and others (Hughes et al., 2017). Along with immediate health effects, ACEs have also been linked to a higher risk of long-term health-harming behaviors, including smoking, sexual risk-taking, damaging alcohol consumption, and drug use (Hughes et al., 2017; Campbell et al., 2016; Song et al., 2020).

It has been suggested that the prolonged biological and psychological stress response to trauma potentially leads to poor emotional regulation and reduced ability to cope with stressors among autistic children (Kerns et al., 2015). Given higher rates of chronic medical conditions and mental health diagnoses compared with their non-autistic peers (Cummings et al., 2016; Hudson et al., 2019; Madden et al., 2016), autistic children may be at increased risk for negative consequences of ACEs. One study of autistic children using the National Survey of Child Health (NSCH) data found that an increase in the number of ACEs is associated with a significant decrease in general health and an increased likelihood of poor mental health, such as anxiety and depression (Rigles, 2017). These findings are in line with findings in the general population and indicate that the effects of ACEs on autistic children’s health can be cumulative, leading to substantially increased risk for those experiencing multiple types of ACEs.

Existing work on ACEs and health has largely relied on cumulative risk, which sums the number of adversities experienced to represent a risk score. Although a risk score can be an effective screening tool to identify children with greater needs for intervention, this approach assumes that all adverse experiences influence health outcomes equally and through the same underlying processes (McLaughlin & Sheridan, 2016). The associations between ACEs and health outcomes may vary across the type and severity of adversity and health outcome. Furthermore, it has been suggested that other childhood adversities that may occur outside the household, such as experiencing racism, witnessing community violence, and encountering bullying, be added to the current definition of ACEs (Cronholm et al., 2015; Finkelhor et al., 2013; Wade et al., 2016; McEwen & Gregerson, 2019). Traditionally measured ACEs were examined primarily for the White, educated, middle-class population (Cronholm et al., 2015). Thus, an investigation into neighborhood adverse exposures may be critical to fully capturing a broader range of adversities experienced across racial and ethnic groups and socioeconomic status (Wade et al., 2016).

A series of prior studies have explored the prevalence of ACEs in families of autistic children (Berg et al., 2016; Kerns et al., 2017; Hoover & Kaufman, 2018). Yet, little attention has focused specifically on transition-age autistic youth (i.e., 12–17 years old). As autistic children develop into adolescence, their needs often shift from behavioral support interventions to mental health support (Ryan et al., 2018). Well-documented challenges and stress experienced during the transition to adulthood (van Schalkwyk & Volkmar, 2017; Taylor & Seltzer, 2011; Friedman et al., 2013) may position those who are adversity-exposed as particularly susceptible to the negative effects of ACEs, which can contribute to suboptimal transition outcomes. One study focusing on transition-aged autistic youth found a significant relationship between prior experiences of trauma and co-occurring psychiatric conditions among autistic youth preparing to exit high school (Taylor & Gotham, 2016). Specifically, 90% of autistic youth with mood and anxiety symptoms had experienced at least one trauma in their lifetime, while 40% of those without mood and anxiety symptoms had traumatic experiences (Taylor & Gotham, 2016).

Overall, understanding the impact of ACEs is essential for targeted screening and prevention of poor health outcomes for autistic individuals, especially for transition-age autistic youth. Elevated adverse experiences in childhood among transition-age autistic youth could contribute to health disparities into and throughout adulthood. In the present study, we used the 2018–2021 National Survey of Children’s Health (NSCH) to identify the prevalence of ACEs in a nationally representative sample of transition-age autistic youth (12–17 years old) in comparison to their nonautistic counterparts. Consistently with previous research (Berg et al., 2016), we expected that autistic youth were more likely to be reported to have individual ACEs and higher cumulative ACEs than their neurotypical counterparts. Second, we sought to estimate the associations between different measures of ACEs (i.e., individual ACEs, cumulative ACEs, and family and community-based ACEs) and different health outcomes. We expected that autistic youth with one or more ACEs overall, family-based, or community-based ACEs would have significantly higher likelihood of experiencing a range of negative physical and mental health outcomes compared to autistic youth who reported no ACEs. Accurate characterizations of this population based on ACEs will help to ensure all transition-age youth get the support they need. This has the potential to improve timely referrals for health and social services and enable successful transitions from high school for all autistic youth.

## Methods

### Data and Study Sample

Four-year NSCH data from 2018 to 2021 were used for this study. Briefly, NSCH is a nationally representative sample survey of children’s health and services administered annually by the US Census Bureau for the Maternal and Child Health Bureau and Health Resources and Service Administration for 50 States and the District of Columbia (Data Resource Center for Child and Adolescent Health, 2016). It includes data on multiple aspects of a child’s health, including physical and mental health and access to health care. It also includes information on the child’s family, school, neighborhood, and other aspects of their social context through a screener questionnaire and an age-based topical questionnaire. The screener questionnaire asks participants to identify all children aged 0 to 17 years living in the household and captures their demographics and special health care needs characteristics. The more-detailed age-based topical questionnaire is then directed to participants if a child lives in the household.

The overall weighted response rates for the NSCH surveys from 2018 to 2021 were 40–43%. A total of 153,632 interviews were completed nationally by parents or caregivers of focal children. For this study, analyses included 2,056 autistic youth aged 12–17 years with available information about their current ASD diagnosis. To identify autistic youth, respondents were asked, “Has a doctor or other health care provider ever told you that this child has Autism or Autism Spectrum Disorder (ASD)?” If they responded yes, respondents were asked whether this child currently had ASD. Youth were excluded from the analysis if they did not have valid responses to ASD history or did not respond to having a current ASD diagnosis. All 2,056 youth had at least one valid response to the ACEs questions (see the section below for ACEs variables in detail).

To compare the prevalence of ACEs, we identified 41,202 neurotypical youth. Participants were excluded from the neurotypical group if caregivers reported that the child had been diagnosed with or currently had a neurodevelopmental disorder or genetic condition by a qualified health care provider, attention-deficit/hyperactivity disorder, learning disability, speech disorder, intellectual disability, developmental delay, Tourette Syndrome, cerebral palsy, Down Syndrome, blood disorder, and other genetic or inherited conditions.

### Measures

#### ACEs Measure

The present study used the NSCH-ACEs, which consists of nine indicators, including extreme hardship due to family income, divorce or separation of parents, parent served time in jail, death of a parent, witnessed domestic violence, living with someone who was mentally ill or suicidal, living with someone with substance abuse problems, treated or judged unfairly due to race/ethnicity, and victim or witness of neighborhood violence (Bethell et al., 2014). Compared to the CDC-Kaiser ACEs questionnaire, the NSCH survey did not assess child abuse and neglect. These nine ACE questions were included based on the observable nature of items and on which caregivers can provide valid reports (Bethell et al., 2017). Each ACE was coded as 0 for no exposure and 1 for exposure. Economic hardship due to family income was captured by questions: “How often it has been very hard to get by on your family’s income?” in which “very often” or “somewhat often” were coded as positive responses and “not very often” or “never” as negative responses.

To capture community-based ACEs, we expanded the NSCH-ACE scale by adding experiences of being bullied and living in an unsafe neighborhood as additional types of ACEs. Bullied experiences were captured by the question: “How often was this child bullied, picked on, or excluded by other children?” in which we deemed negative responses “never” and other responses positive. Living in a safe neighborhood was measured by caregivers’ level of agreement to “This child is safe in our neighborhood”, in which we deemed positive responses “definitely agree” or “somewhat agree” and negative responses “definitely disagree” or “somewhat disagree”.

Affirmative responses across the 11 questions were summed (range: 0–11) to create cumulative ACEs scores, The cumulative ACEs score was also calculated using the 9 indicators included in the NSCH-ACEs scale, which followed the conventional approach in the literature and allowed for prevalence comparability with other studies. We further grouped the 11 ACEs into family-based and community-based ACEs. Specifically, seven ACEs, including extreme hardship due to family income, divorce or separation of parents, parent served time in jail, death of a parent, witnessing domestic violence, living with someone who was mentally ill or suicidal, and living with someone with substance abuse problems were considered family-based ACEs. Four other ACEs were considered community-based ACEs, including treated or judged unfairly due to race/ethnicity, victim or witness of neighborhood violence, bullied experiences, and living in an unsafe neighborhood.

#### Health Outcomes

Outcomes included general health, mental health, and physical health. General health was measured by caregivers’ responses to the questions: “In general, how would you describe [child’s] health? Would you say his/her health is excellent, very good, good, fair, or poor?’ Responses were coded categorically, from 1 (excellent) to 5 (poor). The responses were recoded into excellent/very good (1), good (2), and fair/poor (3).

Mental health measures of the autistic youth included a caregiver-reported current diagnosis of anxiety, depression, and behavioral or conduct problems. Among youth with a current diagnosis of any of the three mental health conditions, respondents were asked to indicate the severity of these conditions (mild, moderate, or severe). According to the current diagnosis status, the three mental health conditions were reported into four levels “No depression/anxiety/behavioral problems” (0), “Mild depression/anxiety/behavioral problems” (1), and “Moderate/Severe depression/anxiety/behavioral problems” (2).

Physical health conditions included the following conditions: allergies, arthritis, asthma, diabetes, epilepsy, heart condition, headaches, and concussion/brain injury. All items were asked about whether (1) a doctor or health care provider had ever told that the child had the condition and (2) whether the child currently had the condition. Children of caregivers who responded “yes” to both these questions were coded as currently having the condition (1), whereas all other children were coded as not currently having the condition (0). The dichotomous variables indicating the presence of a condition were combined to indicate the presence of one or more current physical health conditions. Another variable assessed the focal child’s weight status. Caregivers were asked, “Has a doctor or other health care provider ever told you that this child is overweight?” Overweight was defined as caregivers answering “yes” to this question.

#### Sociodemographic and Clinical Characteristics

Sociodemographic factors included caregiver-reported child and household-level characteristics. Child-level covariates include age in years (12–17 years), sex (male, female), race-ethnicity (Non-Hispanic White, non-Hispanic Black, non-Hispanic Asian, non-Hispanic other race, or Hispanic), health insurance type (private only, public only, both private and public, not specified, not insured). The child’s clinical characteristics included a caregiver-reported current diagnosis of intellectual disability other than ASD and ASD severity (1 = Mild; 2 = Moderate; 3 = Severe).

Household-level socioeconomic characteristics included highest education attainment (no high school diploma or equivalent, high school diploma or equivalent, some college, or college graduate or higher) and federal poverty level (at or below the national level, above the national level). Family income to poverty ratios was calculated using official federal poverty thresholds defined by the U.S. Census Bureau. Due to a large amount of missing data (approximately 16–18% per year), multiply imputed federal poverty level variables created from sequential regression by Census Bureau analysts (U.S. Census Bureau, 2021) were used to account for missingness in poverty level. Other household-level characteristics included residential mobility, which was measured by caregivers’ responses to “How many times has [child] moved to a new address?” Based on the response distribution, responses were categorized into three groups: 0 moves, 1 move, 2 moves, 3 moves, and 4 or more moves. Household size was captured by the answer to the question, “How many people are living or staying at this address?” with responses ranging from 2 to 10.

### Statistical Analysis

NSCH uses a complex survey design to produce nationally representative estimates while accounting for oversampling and nonresponse. Thus, all analyses were conducted with weighted data by using survey weights, strata, and primary sampling units to represent nationally noninstitutional children in the US. Weights were adjusted for the multi-year dataset. All analyses were performed using Complex Samples in SPSS (version 29.0).

Descriptive characteristics of the sample were assessed using both unweighted and survey-weighted proportions. Next, we presented the weighted prevalence of ACEs using the three measures - single ACE, cumulative ACEs (both NSCH-ACE scale and expanded scale), and grouped ACEs (i.e., family or community-based ACEs) among autistic youth. Then the prevalence of ACEs was compared across groups (i.e., autistic youth vs. neurotypical youth) using multinomial logistic regression for individual ACEs and categorical ACEs score and general linear modeling for the total ACEs scores and grouped ACEs, with covariates including age, sex (male as reference), race-ethnicity (non-Hispanic White as reference), insurance type (public insurance only as reference), highest education level in the household (high school degree or lower as reference), federal poverty level (below poverty level as the reference), residential stability (never moved as reference), household size. All ACEs variables had less than 3% missingness. We applied pairwise deletion of observations to handle incomplete information about an ACE. We excluded the observation with a missing value when analyzing that variable, but we still used the observation when analyzing other ACE variables with non-missing values.

Associations between the three methods of ACEs measures and general health, mental health, and physical health were examined using a series of logistic regression models. The adjusted odds ratios (ORs) and 95% CI were obtained to quantify the probability of having health conditions. Specifically, multinomial logistic regression was used for general health and mental health conditions, and binary logistic regression was used for physical health. All regression models were adjusted for sociodemographic and clinical characteristics, including age, sex (male as reference), race-ethnicity (non-Hispanic White as reference), insurance type (public insurance only as reference), highest education level in the household (high school degree or lower as reference), federal poverty level (below poverty level as the reference), residential stability (never moved as reference), household size, co-occurring ID diagnosis, and ASD severity (mild as reference).

Multicollinearity among all predictors was checked using linear regression without considering the complex sample, and the results indicated no severe multicollinearity issue with VIF lower than 2.5 and a tolerance of above 0.4.

## Results

### Sample Characteristics of Autistic Youth

Unweighted and weighted proportions of sample characteristics are presented in Table 1. Of autistic youth aged 12–17, 78.8% were male, and 55.2% were non-Hispanic White. Most of the autistic youth were either covered by public insurance only (33.3%) or private insurance only (46.9%). Among the indicators of household socioeconomic status, 45.8% of the youth had a caregiver with an education of some college or college degree or higher. The percentage of autistic youth living below the federal poverty line was 19.5%. Regarding other household characteristics, the average household size was 4.33, with 40.7% living in a household of five or more people. On average, autistic youth had moved to a new address 3.05 times. Notably, 13.2% of youth had never moved in their lifetime, and 36.9% had moved four or more times.

**Table 1:** The distribution of characteristics of autistic youth (*N* = 2056), NSCH 2018–2021

Variables	Unweighted		Weighted	
	*N*	%	Weighted %	95% CI
Age (M, SD)	14.68	1.66	14.50	14.34, 14.66
Age at first told of ASD (M, SD)	6.44	3.85	6.17	5.81, 6.53
Sex				
Male	1616	78.6	78.8	75.1, 82.1
Female	440	21.4	21.2	17.9, 24.9
Race/ethnicity				
White, non-Hispanic	1487	72.3	55.2	49.5, 60.7
Black, non-Hispanic	133	6.5	11.7	8.9, 15.1
Multiracial/other non-Hispanic	208	10.1	7.1	5.5, 9.0
Hispanic	228	11.1	26.1	20.4, 32.8
Health insurance coverage				
Public only	615	30.3	33.3	28.7, 38.2
Private only	1080	53.2	46.9	41.9, 51.9
Private and public	271	13.4	15.9	12.4, 20.2
Not Insured	63	3.1	3.9	2.6, 5.9
Household education				
Less than high school education	46	2.2	7.4	4.8, 11.3
High school or GED	296	14.4	22.2	17.1, 28.3
Some college or technical school	546	26.6	24.6	20.2, 29.6
College degree or higher	1168	56.8	45.8	41.1, 50.6
Household Size				
Average household size (M, SD)	3.81	1.12	4.33	4.19, 4.48
2	179	8.7	5.2	3.9, 7.0
3	704	34.2	21.8	19.1, 24.8
4	715	34.8	32.2	27.9, 36.9
5 and more	458	22.3	40.7	35.1, 46.9
Federal poverty level				
< 100% FPL	318	15.5	19.5	15.3, 24.6
100–199% FPL	392	19.1	27.9	22.7, 33.7
200–399% FPL	645	31.4	28.6	24.5, 33.2
400+% FPL	701	34.1	24.0	20.9, 27.5
Residential Stability				
Average times of moving (M, SD)	2.63	2.53	3.05	2.77, 3.33
Did not move	422	21.2	16.2	13.2, 19.7
Moved 1 time	399	20.0	17.1	14.2, 20.3
Moved 2 times	281	14.1	12.5	10.3, 15.0
Moved 3 times	319	16.0	17.4	13.7, 22.0
Moved 4 or more times	574	28.8	36.9	31.7, 42.4
Autism Severity				
Mild	1083	53.1	51.4	46.4, 56.4
Moderate	762	37.3	37.2	32.2, 42.4
Severe	196	9.6	11.4	8.2, 15.6
Intellectual disability (Yes)	377	18.5	19.5	15.6, 24.1

Regarding the ASD severity, about half of autistic youth were reported to have mild ASD (51.4%), 37.2% had moderate ASD, and 11.4% had severe ASD. Approximately 19.5% of our sample reported a co-occurring intellectual disability. It is worth noting that this percentage was lower than the population estimates, which suggested that more than one-third of autistic individuals have intellectual disability (Maenner et al., 2023).

### Prevalence of ACEs Between Autistic Youth and Neurotypical Youth

**Fig. 1:**
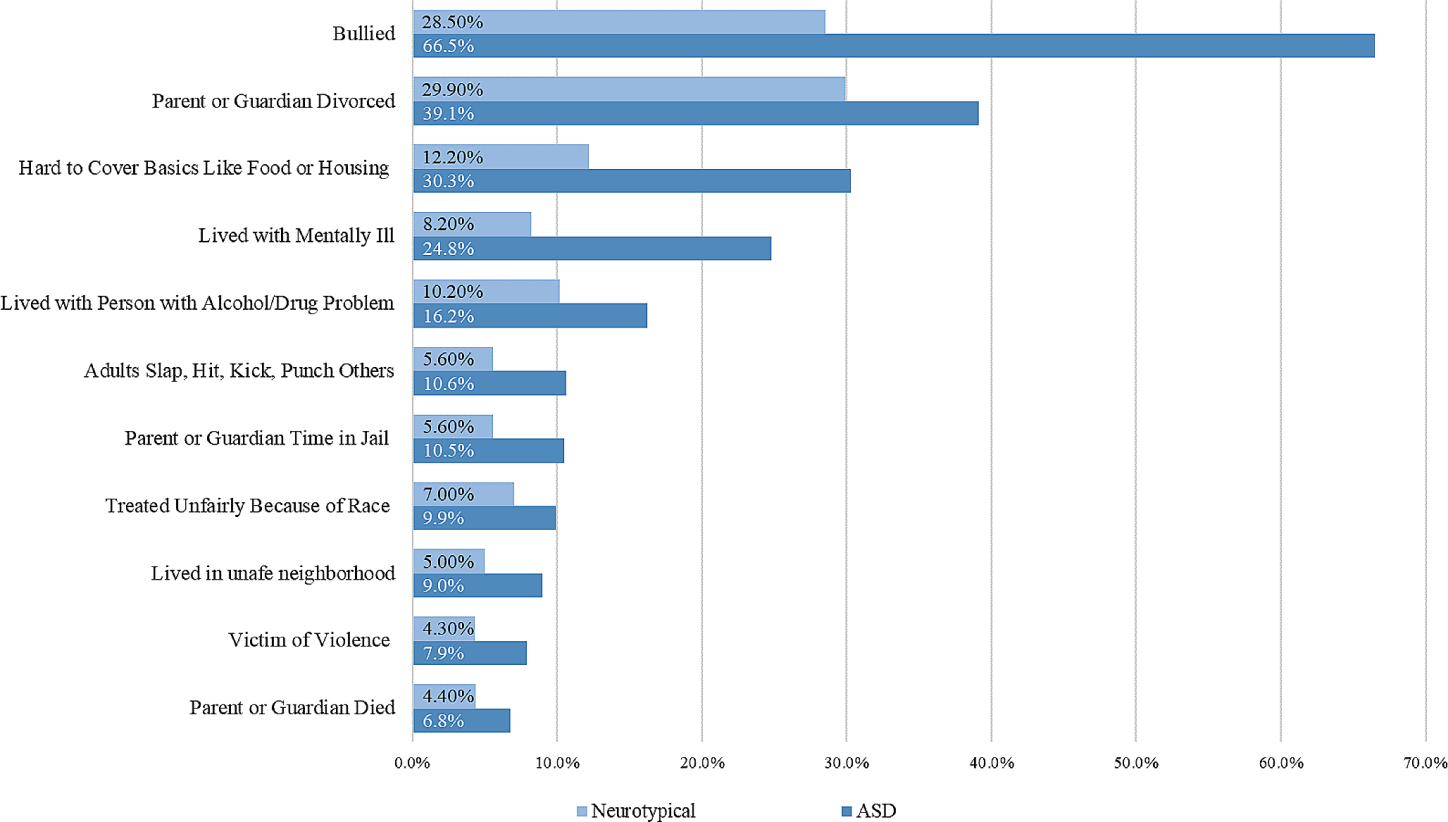
Prevalence (%) of individual ACEs between transition-age autistic youth and neurotypical youth (12–17 years old)

For the prevalence of individual ACEs (see Fig. 1), the most commonly reported ACEs among autistic youth were experiencing bullying in the last 12 months (66.5%, 95%CI [61.4, 71.2]), divorce or separation of parents (39.1%, 95%CI [34.4, 43.9]), extreme hardship due to family income (30.3%, 95%CI [25.1, 36.0]), and living with someone who was mentally ill (34.8%, 95%CI [20.2, 30.1]). In comparison to their neurotypical peers, autistic youth were more likely to be bullied (OR = 5.87, 95%[4.63, 7.45]), live with someone who was mentally ill (OR = 2.75, 95%CI[1.97, 3.84]), experience extreme hardship due to family income (OR = 2.67, 95%CI[2.01, 3.57]), and live in an unsafe neighborhood (OR = 2.18, 95RCI[1.16, 4.09]) were significantly higher. The odds of other individual ACEs were not significantly different among autistic youth and their neurotypical peers.

Regarding cumulative ACEs, prevalence estimates based on the expanded 11-indicator scale showed that 15.7% (95%CI [12.6, 19.5]) of the autistic youth were reported to have no ACEs, and 20.1% (95%CI [15.5, 25.6]) were exposed to four or more ACEs (Table 2). Multinomial logistic regression revealed that autistic youth were more likely to have one or more ACEs compared to neurotypical youth (ORs ranging from 2.26 to 5.95). They also had a significantly higher ACE score than neurotypical youth (1.53 vs. 0.90, b = 0.83, 95%CI[0.62, 1.04]). In order to draw comparisons with other studies that used the NSCH-ACE scale, we also examined cumulative ACEs scores using the NSCH-ACE scale in our study. When using the NSCH-ACE scale, 34.5% (95%CI [30.2, 39.0]) of autistic youth were exposed to no ACE, and 12.4% (95%CI [9.6, 15.9]) experienced four or more ACEs. In comparison to their neurotypical peers, autistic youth were more likely to have one or more ACEs (ORs ranging from 1.40 to 2.52), as well as a significantly higher ACEs score (2.30 vs. 1.23, b = 0.38, 95%CI[0.22, 0.55]).

**Table 2:** Weighted number of total ACE experienced, family-based ACEs, and community-based ACEs

Cumulative ACEs	% of ASD group	% of Neurotypical group	OR^a^ or B^b^	95% CI
Number of Expanded ACEs − 11 Indicator				
0	15.7	39.5	reference	
1	25.6	30.9	2.26	(1.71, 2.99)
2	21.1	14.4	3.61	(2.58, 5.05)
3	17.5	6.7	5.95	(4.06, 8.72)
4 or more	20.1	8.5	5.39	(3.46, 8.41)
Mean score, M(SE)	1.53 (0.08)	0.90 (0.02)	0.83	(0.62, 1.04)
Number of NSCH-ACE – 9 Indicator				
0	34.5	53.5	reference	
1	24.5	25.3	1.40	(1.10, 1.77)
2	20.1	10.0	2.52	(1.79, 3.54)
3	8.4	5.3	1.91	(1.00, 3.62)
4 or more	12.4	5.9	2.35	(1.59, 3.47)
Mean score, M(SE)	2.30 (0.11)	1.23 (0.02)	0.38	(0.22, 0.55)
Family-Based ACEs				
0	38.0	56.7	reference	
1	24.8	24.4	1.36	(1.07, 1.72)
2	17.8	10.0	2.14	(1.51, 3.04)
3+	19.4	8.9	2.28	(1.49, 3.49)
Mean score, M(SE)	1.35 (0.07)	0.78 (0.01)	0.34	(0.19, 0.48)
Community-Based ACEs				
0	30.2	63.1	reference	
1	50.2	30.0	3.83	(3.04, 4.84)
2+	19.6	6.9	6.24	(4.18, 9.33)
Mean score, M(SE)	(0.930.05)	0.45 (0.01)	0.47	(0.38, 0.56)

Note. ORs = Odds Ratio;

^a^ indicates that multinomial logistic regression was used to obtain ORs with categorical ACEs scores as the dependent variables and 0 ACEs as the reference

^b^ indicates that the general linear model was used group differences in total ACEs scores or total grouped ACEs scores

Using the 11 indicators, ACEs were further categorized into family-based and community-based ACEs. Results showed that 38.0% of autistic youth (95%CI[33.6, 42.6]) had no family-based ACE, and 19.4% (95%CI [14.9, 24.9]) were exposed to three or more. Autistic youth were more likely to have one or more family-based ACEs (OR ranging from 1.36 to 2.28) as well as a higher number of family-based ACEs than their neurotypical counterparts (1.35 vs. 0.78, b = 0.34, 95%CI[0.19, 0.48]). For community-based ACEs, about 30.2% (95%CI [25.8, 34.9]) of autistic youth did not experience any community-based ACEs, and 19.6% (95%CI [14.7, 25.6]) experienced two or more community-based ACEs. Autistic youth were more likely to have one community-based ACEs (OR = 33.83, 95%CI[3.04, 4.84]) and two or more community-based ACEs (OR = 6.24, 95%CI[4.18, 9.33]) than their neurotypical counterparts. They also had a significantly higher number of community-based ACEs than their neurotypical counterparts (0.93 vs. 0.45, b = 0.47, 95%CI[0.38, 0.56]).

### Association Between ACEs and Health Outcome Among Autistic Youth

**Individual ACEs and Health.** All logistic regression results of individual ACEs and health outcomes are included in the Supplementary Materials (Figure S1). The association with different health outcomes varied according to ACE type. Among family-based ACEs, exposure to economic hardship was significantly associated with having fair or poor health, moderate or severe anxiety, and being overweight after controlling the covariates. Exposure to parental divorce was only significantly associated with moderate or severe depression and no other health outcomes. Youth who had a parent who served time in jail were more likely to have moderate or severe depression, moderate or severe anxiety, and moderate or severe behavioral problems. Exposure to domestic violence was also associated with moderate or severe depression and moderate or severe anxiety. Living with someone who was mentally ill increased the likelihood of all adverse health outcomes across general health, mental health, and physical health. Living with person with substance problems was also associated with almost all poor health outcomes except for behavioral problems. Parental death was not associated with any health outcomes.

Ammong community-based ACEs, exposure to unfair treatment due to race was only associated with moderate or severe depression and no other health outcomes. Youth who were a victim of neighborhood violence were more likely to have fair or poor health, moderate/severe depression, and moderate/severe anxiety. Exposure to bullying increased the likelihood of having fair or poor health, depression (regardless of severity), anxiety (regardless of severity), and physical health conditions. Last, living in an unsafe neighborhood was only associated with fair or poor health.

**Cumulative ACEs and Health.** Table 3 presents the associations between cumulative ACEs and health outcomes. Transition-age autistic youth more ACEs were more likely to have fair/poor health in general (OR = 1.39, 95%CI [1.16, 1.66]). Regarding mental health, youth with more ACEs were more likely to have mild and moderate/severe depression, mild and moderate/severe anxiety, and moderate/severe behavioral problems (Table 3). When examining physical health, having more ACEs was also associated with a higher likelihood of being identified as overweight by doctors (OR = 1.24, 95%CI [1.09, 1.40]) and to have physical health conditions (OR = 1.14, 95%CI [1.03, 1.27]).

**Table 3:** Commutative ACEs, family-based ACEs, and community-based ACEs, and autistic adult health outcomes: logistic regression analysis

	Cumulative ACE	Family-Based ACEs	Community-Based ACEs
	ORs (95% CI)	ORs (95% CI)	ORs (95% CI)
General Health Status *(Excellent/very good as reference)*			
Good	1.01 (0.91, 1.12)	1.06 (0.90, 1.25)	0.91 (0.65, 1.27)
Fair/Poor	**1.39 (1.16, 1.66)**	1.19 (0.95, 1.50)	**2.00 (1.23, 3.26)**
Mental Health			
*Depression (No as reference)*			
Mild depression	**1.27 (1.12, 1.45)**	1.11 (0.91, 1.35)	**1.80 (1.23, 2.62)**
Moderate/severe depression	**1.59 (1.36, 1.85)**	**1.43 (1.20, 1.69)**	**2.05 (1.43, 2.94)**
*Anxiety (No as reference)*			
Mild anxiety	**1.30 (1.09, 1.56)**	1.05 (0.83, 1.33)	**2.08 (1.35, 3.19)**
Moderate/severe anxiety	**1.49 (1.26, 1.74)**	**1.34 (1.10, 1.62)**	**1.84 (1.33, 2.55)**
*Behavioral Problems (No as reference)*			
Mild behavioral problems	1.07 (0.93, 1.24)	1.09 (0.87, 1.36)	1.04 (0.65, 1.67)
Moderate/severe behavioral problems	**1.21 (1.06, 1.39)**	**1.22 (1.03, 1.45)**	1.19 (0.86, 1.64)
Physical Health			
*Physical Condition*	**1.14 (1.03, 1.27)**	0.97 (0.85, 1.11)	**1.66 (1.26, 2.19)**
*Overweight*	**1.24 (1.09, 1.40)**	**1.40 (1.18, 1.66)**	0.94 (0.65, 1.34)

Note. ORs = Odds ratios; ACEs = Adverse childhood experiences; Bolded ORs represent statistically significant associations. Estimates of cumulative ACEs were calculated in separate regression models from the ones for family-based and community-Based ACEs

**Family-Based and Community-Based ACEs and Health.** The results in Table 3 showed that autistic youth with more family-based ACEs were more likely to have moderate to severe depression (OR = 1.43, 95%CI[1.20, 1.69]) anxiety (OR = 1.34, 95%CI[1.10, 1.62]), and behavioral problems (OR = 1.22, 95%CI[1.03, 1.45]). Autistic youth with more family-based ACEs were more likely to be overweight (OR = 1.40, 95%CI[1.18, 1.66]). However, number of family-based ACEs was not associated with general health status or physical health conditions.

Autistic youth with more community-based ACEs were more likely to have fair or poor health (OR = 2.00, 95%CI [1.23, 3.26]). For mental health, having more community-based ACEs or family-based ACEs were associated with higher likelihoods of reporting depression and anxiety across all severity levels. More community-based ACEs were associated with a higher likelihood of having physical conditions (OR = 1.66, 95%CI[1.26, 2.19]). Community-based ACEs was not associated with behavioral problems or concerns about weight.

**Additional Analysis**. Given the high prevalence of bullying among autistic youth, the associations between community-based ACEs and health outcomes might have been driven by bullying alone. To investigate the specific contribution of each community-based ACEs, we conducted additional logistic regression where four community-based ACEs (e.g., unfair treatment due to race, neighborhood violence, neighborhood safety, and bullying) were treated as separate independent variables. The results are presented in the Supplementary Materials (Table S1). We found that living in an unsafe neighborhood and bullying were significantly associated with higher likelihood of having fair or poor health. For mental health conditions, neighborhood violence was associated with moderate to severe depression and anxiety, and bullying was associated with depression and anxiety across all severity levels. Only bullying showed a significant association with physical health conditions. These findings suggest that while bullying was a predominant and critical predictor of various health outcomes, it was not the sole driver of the associations between community-based ACEs and health outcomes. The significant associations between neighborhood safety and violence with specific health outcomes indicate that these factors also contributed independently to the health challenges faced by autistic youth.

## Discussion

This study sought to examine the prevalence of ACEs and identify health disparities related to ACEs among a nationally representative sample of transition-age autistic youth in comparison to their neurotypical peers. Consistent with prior research (Berg et al., 2016), our findings indicate that autistic youth are at a higher risk for experiencing ACEs than neurotypical youth. Overall, this study confirmed that ACEs were associated with higher odds of adverse health outcomes, including poor general health, mental health, and physical health after controlling relevant demographic and socioeconomic factors. Specifically, this study found that differential health impacts of each ACE. For instance, living with someone mentally or suicidal was significantly associated with more suboptimal health outcomes than other ACEs. Moreover, our study supported the dose-response relationship between ACEs and multiple health outcomes, as individuals with higher ACE counts had an increased likelihood of experiencing fair/poor general health, depression, anxiety, behavioral problems, physical conditions, and being overweight. Importantly, our study underscores the need to understand ACEs in various contexts and their relation to health outcomes. We found that community-based ACEs showed stronger associations with mental health conditions than family-based ACEs, while the likelihood of being overweight was more strongly linked to family-based ACEs.

### Prevalence of ACEs Among Transition-Age Autistic Youth

When using the conventional NSCH-ACE scale, 34.5% of transition-age autistic youth between 12 and 17 years old had *no* exposure to any ACE, a percentage slightly lower than the 38.9% reported in previous research focusing on autistic children aged 3–17 years old (Berg et al., 2016). Notably, this percentage is substantially lower than what has been observed in the neurotypical peers (53.5%) in this study and general population using the same scale in previous studies (e.g., Sacks & Murphey, 2018; Walker et al., 2022). This disparity suggests that transition-age autistic youth likely experience ACEs at disproportionate rates compared to their non-autistic same-age peers. To enhance our understanding of ACEs in this population, our study extended the conventional measurement of ACEs by including two additional items that addressed community adversity - peer victimization and bullying and living in an unsafe neighborhood. Incorporating these factors into the ACEs measure resulted in a decrease in the percentage of transition-age autistic youth who did not have any ACEs, dropping to 15.7%, which was still significantly lower than the neurotypical group (39.5%). This decrease was largely attributed to the high prevalence of being bullied within this group, with a rate as high as 66.5%. This rate is substantially higher than the neurotypical group (28.5%) and the 35.8% rate reported among all adolescents aged 6–17 years in the 2019–2020 NSCH data (Crouch et al., 2023).

Our study findings highlight that both family-based and community-based ACEs are prevalent among transition-age autistic youth. We observed that about 62.0% of the population experienced one or more family-based ACEs, which were significantly higher than the neurotypical group. Likewise, about 50.2% of autistic youth encountered one community-based ACEs, and 19.6% encountered two or more; both rates were significantly higher than the neurotypical group. The notable prevalence of community-based ACEs, which account for multiple dimensions of structural and systematic disadvantages (e.g., being a racial/ethnic minority, neighborhood crime rates), suggest a potential link between adversities in the community settings and those within the family environment. A previous study has found a significant relationship between community violence and household violence (Holtzman & Roberts, 2012). The high prevalence rates in both family and community-based ACE categories underscore the critical need to consider the full spectrum of adversities impacting autistic youth. Future studies should further examine the interplay of family and community-based ACEs, with a focus on understanding how these factors contribute to health disparities observed within the autistic populations and between autistic and non-autistic populations.

### ACEs and Health Among Transition-Age Autistic Youth

Our study conducted a thorough investigation of how individual ACEs, cumulative ACEs, and ACEs across contexts related to various health outcomes in autistic transition-age youth. In our analysis of the association between individual ACEs and health outcomes, we observed that eight out of the 11 ACEs were predictive of depression, and seven were associated with anxiety. This is generally consistent with previous studies examining individual types of ACEs and mental well-being (Merrick et al., 2017; Bellis et al., 2023), indicating a strong influence of ACEs on transition-age autistic youth’s mental well-being. Fewer ACEs showed an association with physical health conditions. Specifically, living with someone who was mentally ill, living with someone with substance abuse problems, and being bullied were linked to physical health issues. Similarly, being overweight was associated with living with someone who was mentally ill and living with someone with substance abuse problems. It is important to note that while our analysis of individual ACEs did not account for the co-occurrence of other ACEs experienced, the evidence suggests that even a single ACE can result in a substantive increase in risk for poor health outcomes. This finding highlights the importance of addressing each ACE in the context of autistic youth’s overall health and well-being.

Our study corroborates the dose-dependent relationship between caregiver-reported ACEs and transition-age autistic youth’s suboptimal health outcomes, including general health status, mental health, and weight. This pattern is in line with literature on the general population (Bomysoad & Francis, 2020; Walker et al., 2022), indicating that ACEs place autistic youth at increased risk for mental and physical health problems at an early age. Moreover, individuals exposed to ACEs often face challenges in emotional regulation and coping strategies, which could lead to social isolation and loneliness (Merrick et al., 2019; Poole et al., 2018; Tzouvara et al., 2023; Forster et al., 2020; Weber Ku et al., 2021). These challenges are often exacerbated by the lack of robust social support, which is crucial for mitigating psychiatric symptoms and managing mental health effectively (Hyland et al., 2019; Kealy et al., 2020; Wang et al., 2018). Consequently, this early exposure may potentially heighten the risk of developing long-term health conditions later in life (Merrick et al., 2019).

However, our analysis of individual ACEs also highlights that not all ACEs contribute equally to health risks. While a higher number of ACEs generally correlates with increased risk, a focus on more ACEs can detract from harms that may arise from exposure to fewer ACEs or even a single ACE (Bellis et al., 2023; Briggs et al., 2021). Thus, it is crucial to recognize the varied impacts of ACEs and how they uniquely impact autistic people. Such recognition is essential in developing tailored interventions that address the multifaceted challenges faced by autistic youth during the transition to adulthood, considering both the number and the nature of ACEs experienced.

One of this study’s major contributions to the literature is the finding of differential associations of family and community adversity with health outcomes in transition-age autistic youth. Specifically, exposure to more community-based ACEs increased the likelihood of having fair or poor general health, depression, anxiety, and one or more physical conditions. Family-based ACEs had a more robust relationship with moderate or severe mental health conditions and being overweight. These findings suggest that interventions that support family and community wellness have the potential to improve the health and longevity of autistic individuals. More specifically, school-based interventions that target bullying and support the inclusion of autistic transition-age youth may improve the overall physical and mental health of this community.

Our research also emphasize the need for future research to move beyond traditional ACEs measures to more holistically examine the effects of ACEs experienced by autistic individuals in different settings (e.g., home, community, and broader society) (Cronholm et al., 2015; Finkelhor et al., 2013). Screening for community-specific ACEs, including community violence, will be particularly essential for capturing the full spectrum of adversity experienced by autistic individuals. For instance, research suggests community violence disproportionately impacts Hispanic and Black youth from low socioeconomic backgrounds (Estrada-Martinez et al., 2013; Giovanelli & Reynolds, 2021). With that, expanding ACEs measures to more fully illustrate the compounding impact of institutional racism and ableism on individuals marginalized by race, ethnicity, and ability is an important direction for future research.

### Limitations

These findings should be considered in light of several limitations. First, as a cross-sectional study, it is not possible to determine the true causal order of ACEs and health status. However, since the NSCH asks about lifetime ACE exposure and current health status, there is some indication that temporality is implied, such that ACEs preceded the measured health outcomes. Nevertheless, temporality is a necessary but not sufficient condition for establishing causality, and we have been careful not to suggest that these findings are causal. Second, caregiver reports might have incomplete knowledge of children’s traumatic experiences or under report socially undesirable events, such as parent incarceration or drug abuse. However, NSCH seeks to mitigate the response bias through its careful weighting structure. Additionally, items on the NSCH undergo extensive testing and purposely avoid asking about physical and sexual abuse, which suggests that such bias is minimal. Third, NSCH did not assess childhood maltreatment, such as abuse and neglect, which has been shown to have more predictive of mental health than household dysfunction (Negriff, 2020). Moreover, binary variables of ACEs did not consider the timing, length of exposure, and severity of the adverse experience. These aspects of ACEs may matter in terms of developing poor health. Future studies on ACEs could benefit from collecting data around the timing of ACEs by asking when they occurred (in cross-sectional design) or monitoring ACEs at regular intervals (in longitudinal design).

### Implication on Policy and Practices

The findings of this study can provide direction for future research on ACEs in the autistic population. First, our finding supports screening for ACEs across both mental health and healthcare settings. Given the high prevalence of ACEs among autistic individuals, screening for ACEs becomes essential to identify youth who may be at greater risk for poor health outcomes. Furthermore, our findings suggest that in order to capture the full spectrum of adversity experienced by autistic youth, ACEs measures must consider the role of individual, family, household, community, and systemic factors, as all can have a negative impact on the health and well-being of this community. Such proactive intervention can help identify and provide support to autistic youth at risk of adverse health outcomes in later life. Future research should continue to investigate causal pathways linking ACEs with mental and physical health issues in autistic individuals across the lifespan – given that challenges associated with these health issues are likely to emerge into adulthood as well.

To fully understand the pathways through which child adversity may result in poor health outcomes in later life, childhood adversity must be accurately classified among various subgroups and within multiple contexts. Many articles have used variants of the original ACEs measures (Felitti et al., 1998; Karatekin et al., 2022), reflecting the drastically different life experiences of children. The findings of this study argue for an expansion of the research on ACEs to include individual, family, and community-level adversities that affect well-being, particularly questions reflecting community ACEs (e.g., community violence, racism). Understanding the ACEs experiences of specific subgroups especially critical given the heterogeneity of the ASD population. For instance, research shows that LGBTQIA + communities experience more ACEs compared to individuals who identify as heterosexual (Anderson & Blosnich, 2013; McCabe et al., 2020). Transgender individuals are more likely to report emotional abuse, emotional neglect, and physical neglect compared to those who identify as cisgender (Schnarrs et al., 2019). Given that many members of the autistic community share these general and sexual identities (McQuaid et al., 2023; Walsh et al., 2018), it is imperative for research to include the experiences of autistic individuals with diverse identities when working to inform policy and intervention practice.

While primary prevention efforts should be targeted at preventing ACEs from occurring, it is equally important to identify protective factors that could reduce the negative impacts of ACEs on youth’s health (Bethell et al., 2019). Service providers are encouraged to adopt ACEs intervention strategies to mitigate the risk of health problems in childhood, which could have long-term impacts on occupational and social outcomes in adulthood. For instance, trauma-informed practices can be used as a therapeutic intervention for those affected by ACEs (Oral et al., 2016; Ranjbar & Erb, 2019). Interventions to help to build supportive and inclusive relationships among family members and communities may help to mitigate the negative long-term effects of ACEs exposures. For instance, a previous study has found that feeling supported by neighbors can help reduce behavioral problems among adolescents aged 12–17 with a prior ACE (Khanijahani & Sualp, 2022). In light of these findings, a comprehensive and inclusive community-based approach that combines prevention, early intervention, and trauma-informed care is paramount to addressing the multifaceted challenges posed by ACEs.

## Conclusion

Our study provides one of the most comprehensive assessments of the interrelationships between ACEs and transition-age autistic youth’s general health, mental health, and physical health. This finding bridges a gap in determining the associations of ACEs with poor health outcomes for transition-age autistic youth and provides a better understanding of how ACEs at both family and community level can present risks to health for this population. Our results provide the prevalence of ACEs for four years of nationally representative data, providing robust estimates to inform decisions in programming and policies. We found that ACEs were common among transition-age autistic youth, with experiences of bullying most frequently reported. Our findings could contribute to how we understand health disparities in autistic individuals and what contributes to their high rates of poor health outcomes in later life. Thus, we must invest in efforts to promote family and community well-being alongside early screenings for ACEs in order to improve the health trajectories of autistic youth.
